# Spatially Resolved Distribution of Fe Species around Microbes at the Submicron Scale in Natural Bacteriogenic Iron Oxides

**DOI:** 10.1264/jsme2.ME17009

**Published:** 2017-09-27

**Authors:** Hiroki Suga, Sakiko Kikuchi, Yasuo Takeichi, Chihiro Miyamoto, Masaaki Miyahara, Satoshi Mitsunobu, Takuji Ohigashi, Kazuhiko Mase, Kanta Ono, Yoshio Takahashi

**Affiliations:** 1 Department of Earth and Planetary Systems Science, Graduate School of Science (DEPSS), Hiroshima University Higashi-Hiroshima, Hiroshima 739–8526 Japan; 2 Project Team for Development of New-Generation Research Protocol for Submarine Resources, Japan Agency for Marine-Earth Science and Technology (JAMSTEC) Natsushima, Yokosuka, Kanagawa 237–0061 Japan; 3 Institute of Materials Structure Science, High-Energy Accelerator Research Organization (KEK) Oho, Tsukuba, Ibaraki 305–0801 Japan; 4 Department of Materials Structure Science, SOKENDAI (The Graduate University for Advanced Studies) 1–1 Oho, Tsukuba, Ibaraki, 305–0801 Japan; 5 Department of Earth and Planetary Science, Graduate School of Science, The University of Tokyo Bunkyo-Ku, Tokyo 113–0033 Japan; 6 Department of Environmental Conservation, Graduate school of Agriculture, Ehime University Tarumi, Matsuyama, Ehime 790–8577 Japan; 7 UVSOR facility, Institute for Molecular Science Myodaiji, Okazaki 444–8585 Japan

**Keywords:** iron oxides, iron speciation, bacteriogenic/biogenic iron oxides (BIOS), scanning transmission X-ray microscopy (STXM), near edge X-ray absorption fine structure (NEXAFS)

## Abstract

Natural bacteriogenic iron oxides (BIOS) were investigated using local-analyzable synchrotron-based scanning transmission X-ray microscopy (STXM) with a submicron-scale resolution. Cell, cell sheath interface (EPS), and sheath in the BIOS were clearly depicted using C-, N-, and O- near edge X-ray absorption fine structure (NEXAFS) obtained through STXM measurements. Fe-NEXAFS obtained from different regions of BIOS indicated that the most dominant iron mineral species was ferrihydrite. Fe(II)- and/or Fe(III)-acidic polysaccharides accompanied ferrihydrite near the cell and EPS regions. Our STXM/NEXAFS analysis showed that Fe species change continuously between the cell, EPS, and sheath under several 10-nm scales.

Fe oxides are ubiquitously present on the Earth’s surface (19). The behaviors of cations and anions are affected by Fe oxides because of their large surface area and high absorptive characteristics (7). The formation of some Fe oxides has been associated with microbial activities. Natural Fe oxides are composed of inorganic Fe oxides (29) and biogenic Fe oxides that originate from the metabolites of Fe-oxidizing bacteria (8, 9). The latter are called bacteriogenic (or biogenic) iron oxides (BIOS) (*e.g.*, 10, 13, 19).

BIOS are a mixture of fine Fe oxides with organic matter such as microbial cell bodies, extracellular polysaccharides (EPS), tubed sheaths, and twisted stalks (8, 10–12, 30, 31). Therefore, BIOS have micron-scale heterogeneity when observed microscopically. In previous studies, a scanning transmission X-ray microscopy (STXM)-based near edge X-ray absorption fine structure (NEXAFS) analysis (hereafter STXM/NEXAFS) was performed on several BIOS samples in order to characterize the chemical species of C, N, O, and Fe for each component (4–6). The findings obtained indicated that Fe chemical species within BIOS do not markedly vary. In contrast, the Fe(III) that precipitates in the vicinity of Fe-oxidizing bacteria consists of various Fe mineral species, suggesting the heterogeneity of Fe chemical species in natural Fe mat samples (21). Therefore, studies on Fe chemical species at the micron scale using STXM/NEXAFS have become an important technique for understanding Fe mineral species in specific domains of samples such as microbe-metabolite interfaces.

The main aim of the present study is to elucidate the adsorption mechanisms of trace elements adsorbed on local areas (*e.g.*, cell walls) within natural BIOS such as initiated by bulk-analysis results in Takahashi *et al.* (32). Here, we also investigated Fe chemical species on/in each component including microbial cells, EPS, and metabolites at several tens of nanometer scales: as the preliminary step.

In the present study, BIOS were collected from Budo pond in the Higashi-Hiroshima campus of Hiroshima University (34° 24′ 03. 500″ N, 132° 42′ 47. 200″ E). Fe(II)-rich groundwater is constantly supplied into the pond. Fe precipitates (approximately 15 cm in depth/thickness) containing BIOS form around the outlet of groundwater (16, 17, 33). The presence of the Fe(II)-oxidizing bacteria, *Gallionella* spp. and *Leptothrix* spp., was confirmed by a 16S rDNA analysis in previous studies. Throughout the year, water temperature ranges between 15 and 22°C, pH between 6.2 and 6.5, dissolved Fe(II) concentrations at approximately 290 μM, and dissolved oxygen concentrations (DOC) between 29 and 299 μM (16). Variations in DOC may be related to the degree of mixing of anaerobic groundwater and aerobic pond water.

BIOS samples were collected into 50-mL Falcon Conical Centrifuge Tubes (Becton Dickinson), transferred to the Photon Factory in Tsukuba, Japan, and analyzed using STXM/NEXAFS within 36 h in order to minimize the oxidation of Fe minerals and organic matter alterations. Twenty microliters of the sample was dropped onto a silicon grid with a 50-nm-thick Si_3_N_4_ membrane (Silson), and then air-dried under ambient conditions for 20 min. In the present study, the compact STXM system installed at BL-13A in the Photon Factory, KEK (Tsukuba, Japan) was used (34). The STXM system installed at BL-4U in UVSOR (Okazaki, Japan) was also employed to obtain NEXAFS for N K-edge (23). All NEXAFS spectra including reference materials were taken by the image stacking method (15), in which an image of 20 by 30 dimensions (at least) of the selected sample area was obtained by initially scanning the sample with a fixed energy. Energy was then changed and the same area was scanned again. This process was repeated with changes in energy. STXM measurements were performed within the energy region of the K-edges of C (between 280 and 300 eV), N (between 395 and 425 eV), and O (between 520 and 550 eV) and L-edge of Fe (between 700 and 730 eV). C-NEXAFS spectra were obtained for every 0.1- to 0.5-eV step, while N-, O-, and Fe-NEXAFS were obtained for every 0.1- to 1-eV step. Sequential images were combined as a stacked dataset. In the present study, the size of the pixels in all images was fixed at 50 nm. In addition, the spatial resolution of this analysis was approximately 40 nm at the C, N, and O K-edges, and 50 nm at Fe L-edge. Regarding standard materials, ferrihydrite as a representative Fe oxide standard was made as described by Schwertmann and Cornell (25). Fe(II)- and Fe(III)-alginates as acidic polysaccharide standards were also prepared following Mitsunobu *et al.* (22). Standard spectra were obtained with energy steps between 0.1 and 0.5 eV. The extraction of images and spectra from energy-dependent image data was conducted using aXis2000 software (A. P. Hitchcock, the aXis2000 analysis package is written in an Interactive Data Language [IDL], http://unicorn.mcmaster.ca/aXis2000.html). Thus, a principal component analysis (PCA) and cluster analysis were conducted using Mantis software (http://spectromicroscopy.com). The XAFS analysis software, REX2000 (Rigaku, Japan) was used to normalize NEXAFS spectra. In addition to STXM measurements, secondary electron (SE) images were obtained using a scanning electron microscope (SEM: KEYENCE VE-9800) equipped with an X-ray energy dispersive spectrometer (EDS: EDAX, AMETEK Co., Ltd.). Peak fitting was conducted by the multipack fitting program in IGOR Pro (WaveMetrics, Inc.).

Fig. 1a shows C and Fe distributions in BIOS collected from Budo pond. High Fe containing sheath-like tubed filament structure is probably sheath (hereafter, sheath) from the fluorescence microscopy observation (Fig. S1) and C-NEXAFS feature (discussed below: Fig. S2). In addition, previous studies for sheath supported our results (12, 16, 17, 33). A high C content rod-shaped portion was observed on the surface of the sheath. The morphology of this portion was similar to the collapsed microbe-like structure observed by scanning electron microscopy (Fig. S3) (4, 6). C-, N-, and O-NEXAFS obtained by our STXM measurements indicated that the high C content rod-shaped portion was the cell of a microbe (hereafter, cell), and the cell-sheath interface was EPS-like matter. Fig. 1b shows C-NEXAFS for the cell-like region and sheath-like regions. Peaks at 285.2, 288.2, and 288.6 eV were confirmed in both spectra, as well as the shoulder at 287.3 eV (Fig. 1b). We summarized the functional groups and transition corresponding to each peak of C, N, and O in Table S1 (2, 4–6, 18, 20–22, 26–28). Each peak at 285.2, 288.2, and 288.6 eV originated from a protein (1s→π*C=C), protein (1s→π*C=O), and acidic polysaccharide carboxyl (1s→π*C=O), respectively (Table S1). In addition, the shoulder at 287.3 eV indicated a lipid (1s→3p/σ*C-OH). The protein peak at 288.2 eV was dominant in the cell region, while the polysaccharide peak at 288.6 eV was dominant in the sheath spectrum. This spectrum feature was consistent with previous findings (2, 4–6, 18, 20). The spectrum obtained from the cell-like region had a peak at 289.3 eV, indicating a major transition peak of DNA (1s→3p/σ*C-O) (Fig. S2). The sheath portion did not show a peak at this energy. However, the spectrum was noisy and the peak of DNA was only clearly confirmed from the spectrum obtained from the cell region. Consequently, the accurate identification of DNA was difficult in the present study. In addition, the major organic content in the sheath was suggested to be polysaccharides by the C-NEXAFS analysis, which is similar to the cultural and environmental stalk (4–6). C-NEXAFS of the cell-sheath interface suggested the presence of EPS-like matter from the peak features at 285.2, 288.2, and 288.6 eV, and the shoulder of 287.3 eV, as well as the high content of polysaccharides, which is consistent with previous findings (*e.g.*, 20, 22). N-NEXAFS spectra were obtained at the same domains of C-NEXAFS (Fig. 1c). N-NEXAFS obtained from the cell-like region had a peak at 401.2 eV (1s→π*N-C=O), indicating the presence of a protein with an EPS and DNA (2, 6, 27, 28). In addition, a weak peak at 399.0 eV suggested the transition of 1s→π*N=C (DNA). The shoulders at 399.9 eV and 402.2 eV also indicated 1s→π*N-C (DNA) and 1s→π*N-C (Amine/Amino N with EPS and DNA), respectively. Furthermore, a broad peak greater than 405.0 eV reflected transitions of amino acids with the mixing of aliphatic, alcohol, carboxylic, and aromatic resonances (a protein with lipids, EPS, and DNA) (2, 27, 28). However, clear identification was difficult, particularly for weak peaks and shoulders, because the signal-to-noise ratios (S/N ratios) of N-NEXAFS spectra were poor. The sheath did not exhibit these peaks. The O K-edge NEXAFS of the cell-like region and sheath portion were also taken (Fig. 1d). Both regions had similar NEXAFS features at the high-energy (greater than 535.0 eV) region, reflecting the O-Fe bond of iron oxides/mixing of the O-C bonds of organics (6, 21, 26). However, a difference was observed at the low-energy region (up to 535.0 eV). In the low-energy region, t_2g_ and e_g_ peaks corresponding to iron oxides (O2_p_-Fe3_d_) (26) were confirmed at 529.2 eV and 530.5 eV, respectively. The energy of the d orbitals in a spherical environment (a free atom) was degenerating. However, the d orbitals of transition metal elements were split into a triply degenerate set (t_2g_) at lower energy and a doubly degenerate set (e_g_) at higher energy separated by splitting energy (Δ_O_) in octahedral (O_h_) crystal fields. The energies of the e_g_ orbitals are known to be higher than those of the t_2g_ orbitals (1, 36), which leads to the lower energy of the peak assigned to t_2g_ than that of the peak e_g_ in Fig. 1d. Detailed research on the O-NEXAFS of Fe oxides has already been conducted by Chan *et al.* (6), and the findings obtained confirmed the e_g_ and t_2g_ peaks from all O-NEXAFS of Fe oxides regardless of crystallinity (such as poorly 2-line ferrihydrite, and well-crystallized goethite and akaganeite). The reason for the decrease in this peak height was not provided by Chan *et al.* (6). In addition to e_g_ and t_2g_, O-NEXAFS of the cell-like region had a peak corresponding to a protein (or polysaccharide) at 532.7 eV (6, 21, see table S1).

Fig. 1e shows a functional group image of the microbe (cell+EPS-like; See Fig. 2a). The microbe and sheath both showed a similar distribution of C functional groups. The functional group image also revealed that the microbe was richer in C than the sheath. In addition, a globule-like structure was observed in the sheath (Fig. 1e, f, and S5b, c), which resembled the inner/outer globular surface structure related to C and fine Fe oxides composed of a thin fibrous surface structure observed by STEM/EELS in Suzuki *et al.* (30) (11, 12, 24, 31). The inner/outer globular surface structure was mainly composed of polysaccharides with proteins and lipids, which is consistent with previous findings (8, 11, 12). A similar structure, based on its elemental composition and structure size/shape, was reported by Suzuki *et al.* (30) and Sakai *et al.* (24) using SEM mapping, as well as metal deposition related to outer/inner sheath structures by Furutani *et al.* (11, 12). STXM-composited images showed the distribution of C functional groups in the sheath, as shown in Fig. 1e in the present study. Furthermore, we confirmed that this structure was not an artifact by analyzing an olivine thin film with a uniform thickness prepared by the focused ion beam system SMI3200 (Hitachi High-Tech Science Corp./Seiko Instruments Inc. [SII]), which did not show any spotted structures. STEM/EELS and STXM/NEXAFS are powerful tools for spatially resolved chemical analyses based on the interactions of electrons/X-rays and elements, respectively. Nevertheless, STEM/EELS are superior in terms of spatial resolution because of the diffraction limit of FZP in STXM. On the other hand, STXM/NEXAFS (synchrotron-based) has better energy resolution. In addition, STXM/NEXAFS has an advantage in terms of radiation damage for wet/soft samples analyses (14).

Fe L-edge NEXAFS was obtained from the cell, EPS-like, and sheath portions to investigate Fe chemical species on/at each component (Fig. 2a). The Fe L-edge spectrum contained L_2_ (2p_1/2_→3d_3/2_, 721.0–727.0 eV) and L_3_ (2p_3/2_→3d_3/2_3d_5/2_, 706.0–712.0 eV) absorption edges, which originated from the spin-orbit interaction of the 2p core hole in the 2p^5^3d^5^ (Fe[II]) and 2p^5^3d^6^ (Fe[III]) cases (3, 26, 35). L_2,3_ multiple structures clearly reflected valence state changes (chemical species), site symmetry, and crystal field strength related to e_g_ and t_2g (_3, 26, 35). However, difficulties were associated with understanding the Fe-NEXAFS spectral features of environmental samples from only the logical spin configurations including site symmetries and crystal fields because natural samples are not pure crystals (containing Fe in other materials including Fe-organic matter, Fe carbonate, and clay minerals). Therefore, we attempted to identify the Fe-NEXAFS of the cell, EPS-like, and sheath portions using comparisons with standard materials. All Fe-NEXAFS spectra were background-subtracted by REX2000. Based on comparisons with the spectra of reference materials, all NEXAFS obtained from the cell, EPS-like, and sheath portions were similar to that of ferrihydrite, characterized by peaks at 707.6 eV (t_2g_) and 709.2 eV (e_g_) (Fig. 2a). These results indicate the presence of short-range ordered Fe oxides at the cell, EPS-like, and sheath portions, which is consistent with Fe speciation in the bulk analysis (22). In addition, Fe-NEXAFS in this sheath-like portion was homogeneous, and not related to globule-like structures, indicating that the chemical compound within the sheath region was homogeneous (Fig. S6). A PCA and cluster analysis for similar region of Fig 1e confirmed variability in Fe-NEXAFS under the few tens of nanometers scale (Fig. S6). The structure of the e_g_ peak around 709.2 eV showed that (i) peak height was less in the EPS-like and microbe than in ferrihydrite (Table 1a), and (ii) the peak structure of the cell appeared to split at the e_g_ region (Fig. 2b, S7), implying the presence of an additional component in Fe mineral species rather than spin orbital interactions. The decrease observed in the e_g_ peak was also confirmed in the low energy region of O K-edge NEXAFS (Fig. 1d, S4, and table S2), and this was attributed to variations in Fe mineral species at each component. This spectral heterogeneity was not confirmed in Fe-NEXAFS obtained from the total region (Fig. 2a). Chan *et al.* (4, 6) indicated that the dip between t_2g_ and e_g_ peaks of O- and Fe-NEXAFS becomes deeper with better crystallinity, considering with Fe-NEXAFS and XRD results. However, Fe-bonding organic material standards, including Fe-alginate, were not examined by Chan *et al.* (5). Therefore, this dip feature is beyond the scope of the present study. In addition, the S/N ratio of O-NEXAFS spectra was not sufficiently high to accurately discuss the dip feature. Comparisons of Fe-NEXAFS obtained from the cell with reference materials implies that the prominent peak of Fe(III)-alginate at 708.9 eV may be superimposed with that of ferrihydrite at 709.2 eV (the fitting result showed 709.15 eV) in NEXAFS of the microbe (Fig. 2b; Table 1c), suggesting the greater contribution of acidic polysaccharides on the microbe than other regions. In other words, the fraction of ferrihydrite in the microbe is lower than those of the sheath and EPS, which is responsible for the decrease in the e_g_ peak of O- and Fe-NEXAFS. Fe(III)-alginate may be preferentially coordinated in the microbe and EPS-like regions in natural BIOS samples. A similar condition, in which some metabolic processes in Fe-oxidizing bacteria are located in close proximity to the sites of polymer secretion (including EPS-like matter), was already predicted and suggested by Chan *et al.* (4, 6). In addition to Fe(III)-alginate, Fe(II)-alginate (at 707.2 eV) has been suggested to exist in the cell (Fig. 2b) because the heights of spectra around 707.2 eV were higher in the EPS and cell regions than in the sheath region, suggesting the contribution of the Fe(II) peak of Fe(II)-alginate. Therefore, the direct detection of Fe(II) adsorbed on Fe oxyhydroxide (including ferrihydrite), which catalyzes iron oxidation, may be possible (4). In addition, the Fe(III)/Fe(II) ratio appeared to continuously change from the sheath to the cell (Table 1b). These results indicate the presence of Fe(III)-alginate and Fe(II)-alginate (or fine ferrihydrite in EPS-like matter) in our STXM/NEXAFS analysis despite the unclear identification of EPS between the cell and sheath regions.

Our STXM analyses allowed us to successfully depict the submicron-scale spatially-resolved distributions of various Fe species in the cell, EPS-like, and sheath regions of natural BIOS samples collected from Budo pond. Fe(III) precipitates in BIOS samples were mainly Fe oxides associated with the EPS-like or sheath region. NEXAFS spectral features indicated that some Fe(II) and Fe(III) ions directly bonded to EPS; *i.e.*, the Fe-EPS complex formed on the cell and EPS-like regions. STXM/NEXAFS revealed the localized distribution of Fe chemical species, which had been overlooked in previous studies conducted using a bulk analysis. A STXM/NEXAFS technique may be applied in order to elucidate adsorption mechanisms at local areas (*e.g.*, cell surface) in BIOS related to chemical species/the distribution of trace elements. A combined-STXM and FISH method has already been used to investigate the adsorption mechanisms of each microbe species (*e.g.*, 20, 22). Our site-specific STXM/NEXAFS analysis will become a key analytical tool for the investigation of metabolism mechanisms used by uncultivated microbes for chemical speciation.

## Supplementary Material



## Figures and Tables

**Fig. 1:**
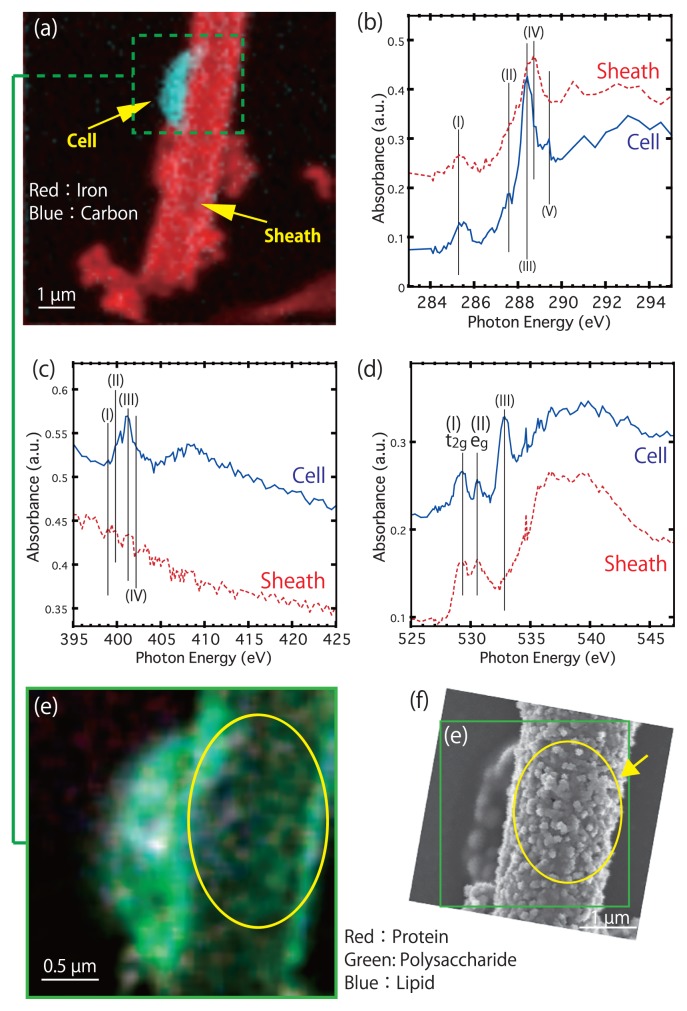
(a) Composition map of C (blue) and Fe (red). Yellow arrows indicate the microbe (cell+EPS) and sheath. (b) C-NEXAFS obtained from rod-shaped (cell) and metabolite (sheath) portions. (c) N- and (d) O-NEXAFS of the cell and sheath. Each spectrum obtained from approximately the same region of (b). The transition of peaks (I)–(V) in (b), (c), and (d) was indicated in Table S1. (e) RGB image of functional groups of C. Proteins (red), polysaccharides (green), and lipids (blue) were detected. A yellow-circled region indicates the globule-like structure on the sheath, which may be related to the inner/outer globular structure. (f) Secondary electron image around the area of (e) obtained by SEM. The region of (e) was indicated as a green square. The outer globule-like structure of the sheath was shown in the yellow-arrowed and yellow-circled area.

**Fig. 2:**
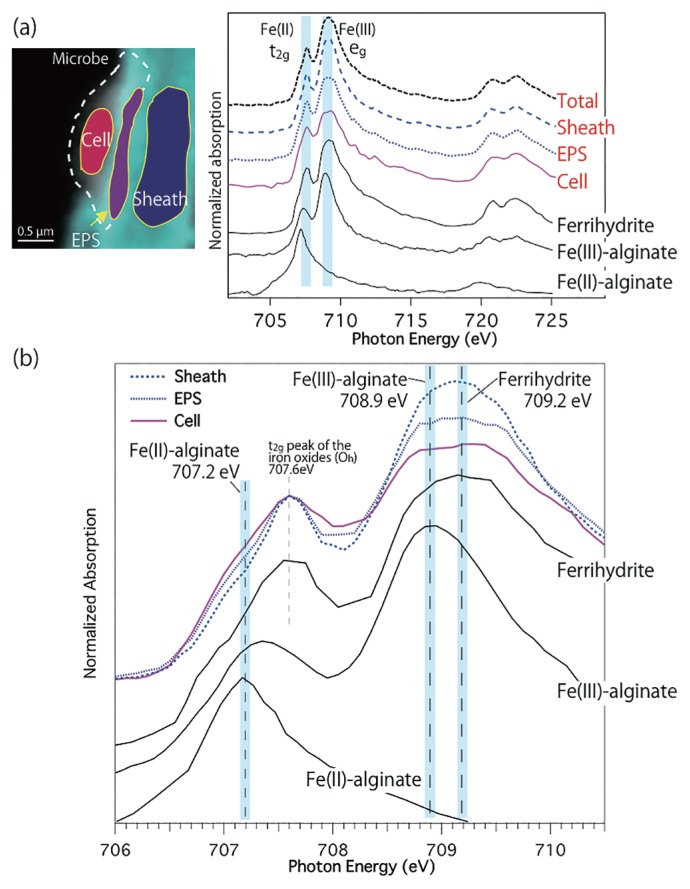
(a) Fe L-edge NEXAFS obtained from cell, EPS, and sheath portions. A total spectrum was obtained from the total colored area of Fig. 1(e), including the cell, EPS, and sheath portions. Ferrihydrite, Fe(III)-alginate, and Fe(II)-alginate are also shown as standard spectra. (b) Expansion graph of Fe L-edge NEXAFS obtained from the cell, EPS, and sheath portions (same spectra of [a]). These spectra were normalized to t_2g_ peak intensity (at 707.6 eV).

**Table 1:** (a) Fe(III) peak heights from t_2g_ peak intensity-normalized NEXAFS. (b) Fe(III)/t_2g_ peak height ratios for each component. (c) Main contributing peaks at the e_g_ peak region from the peak separation fitting shown in Fig. S7.

	(a)	(b)	(c)
Sample	Fe(III) (Peak height)	Fe(III)/t_2g_	Contributing peaks at the Fe(III) peak (From Fig. S4)
Sheath	80.79	1.60	709.10 eV
EPS	70.92	1.40	708.75, 709.37 eV
Cell	63.35	1.25	708.72, 709.30 eV
Ferrihydrite	75.36	1.49	709.15 eV
