# Supplementary material for: Spatially Resolved Distribution of Fe Species around Microbes at the Submicron Scale in Natural Bacteriogenic Iron Oxides
Source: Microbes Environ. 2017 Sep 27;32(3):283–7. doi: 10.1264/jsme2.ME17009 (PMC5606699; doi:10.1264/jsme2.ME17009)
Supplement: Supplementary file 1 [file 32_283_s1.pdf]

Supplementary materials for

"Spatially Resolved Distribution of Fe Species around Microbe at Submicron Scale in the Natural Bacteriogenic Iron Oxides"

Hiroki Suga[1*], Sakiko Kikuchi[2], Yasuo Takeichi[3,4], Chihiro Miyamoto[5], Masaaki Miyahara[1], Satoshi Mitsunobu[6], Takuji Ohigashi[7], Kazuhiko Mase[3,4], Kanta Ono[3,4], and Yoshio Takahashi[1,3,5]

[1] Department of Earth and Planetary Systems Science, Graduate School of Science (DEPSS), Hiroshima University, Higashi-Hiroshima, Hiroshima 739-8526, Japan; [2] Project Team for Development of New-Generation Research Protocol for Submarine Resources, Japan Agency for Marine-Earth Science and Technology (JAMSTEC), Natsushima, Yokosuka, Kanagawa 237-0061, Japan; [3] Institute of Materials Structure Science, High-Energy Accelerator Research Organization (KEK), Oho, Tsukuba, Ibaraki 305-0801, Japan; [4] Department of Materials Structure Science, SOKENDAI (The Graduate University for Advanced Studies), 1-1 Oho, Tsukuba, Ibaraki, 305-0801, Japan; [5] Department of Earth and Planetary Science, Graduate School of Science, The University of Tokyo, Bunkyo-Ku, Tokyo 113-0033, Japan; [6] Department of Environmental Conservation, Graduate school of Agriculture, Ehime University, Tarumi, Matsuyama, Ehime 790-8577, Japan; and [7] UVSOR facility, Institute for Molecular Science, Myodaiji, Okazaki 444–8585, Japan

* Corresponding author. E-mail: hiro-suga@hiroshima-u.ac.jp; Tel: +81-82-424-7459; Fax: +81-82-424-0735.

Figure S1

(a) (b)

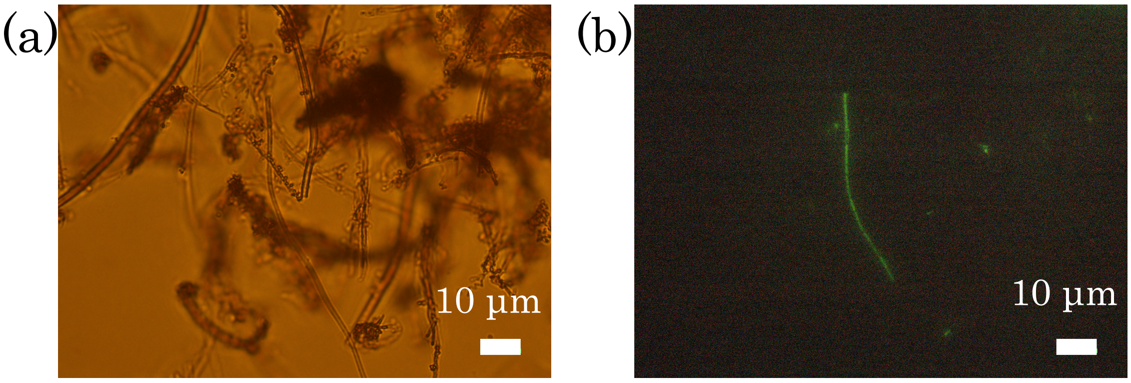

Figure S1. (a) Representative optical microscopy image of the Budo pond Fe sediment containing BIOS. Sheath-like materials were confirmed in the sample, consisted with previous reports (16, 34). (b) Fluorescent image of the same region of (a) by a fluorescence microscopy (BX60, Olympus). Green-fluorescent in (b) showed the presence of rod-shape living-bacteria cell (probably related to *Lepthrix* spp.) in the some of the sheath-like portion by SYTO 13 (Thermo Fisher Scientific) stein under the dark condition for 5 minute. In addition, most sheath-like portions were empty or filled with dead cell, judging from the lack of fluorescent emission.

Figure S2

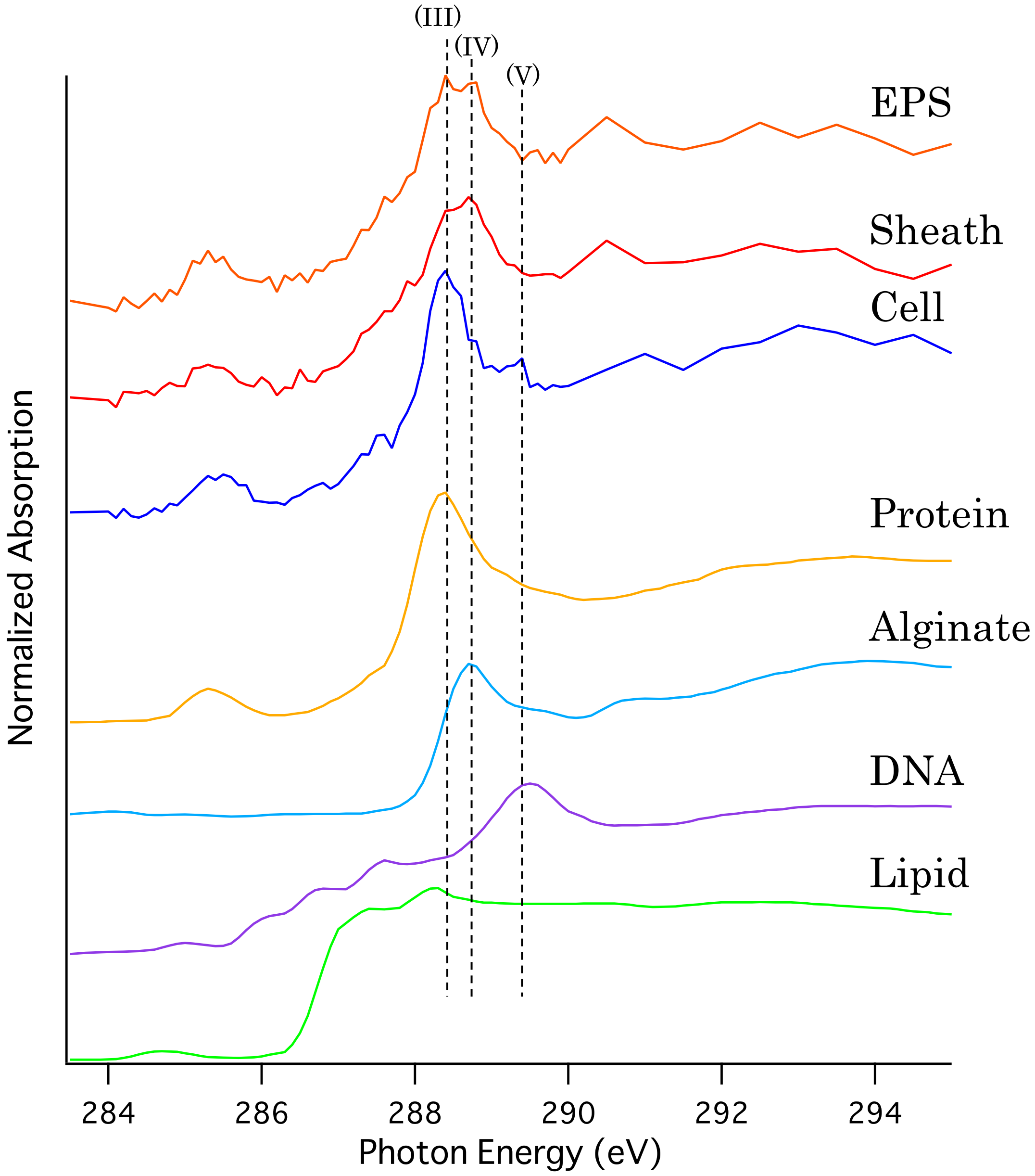

Figure S2. C-NEXAFS obtained from cell, EPS, and sheath portions with reference spectra (from Mitsunobu et al., 2016). Protein (288.2 eV) was dominant in the cell portion. In contrast, alginate (288.6 eV) was dominant in the sheath portion. Both protein and alginate was confirmed at the EPS-like portion, which is consistent with Figure 1 (e). DNA (289.3 eV) was only confirmed in the cell portion.

Figure S3

(a)

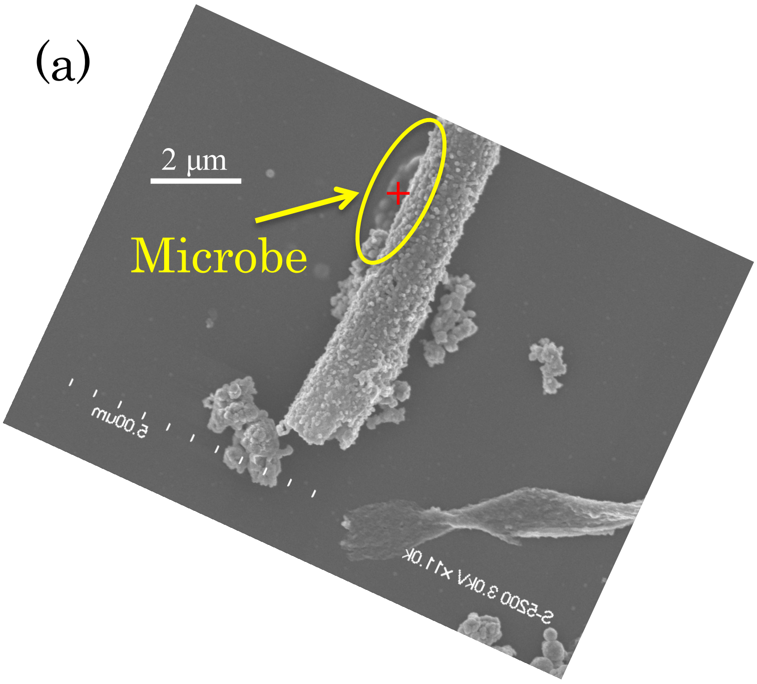

(b)

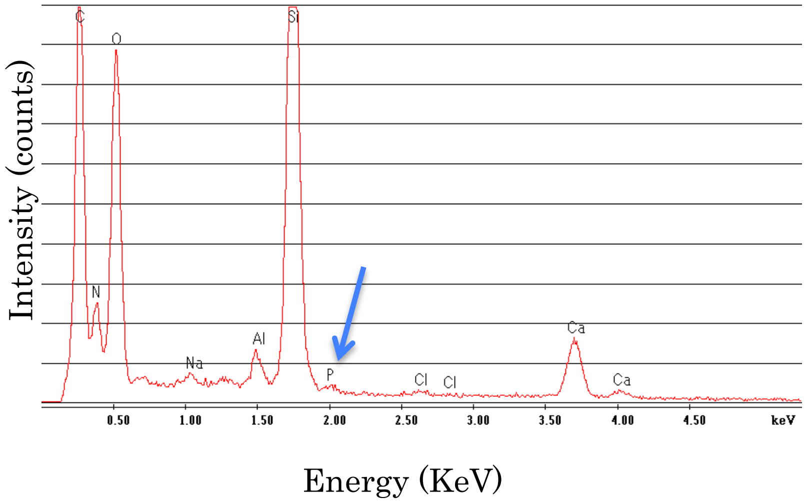

Figure S3. (a) Secondly electron (SE) image of BIOS samples studied here. The collapsed microbe and sheath (corresponding to figure 1(a)) are circled in yellow. (b) X-ray fluorescence spectrum collected at the red-cross point of the microbe in Figure S1(a). The microbe includes a small amount of phosphorus.

Figure S4

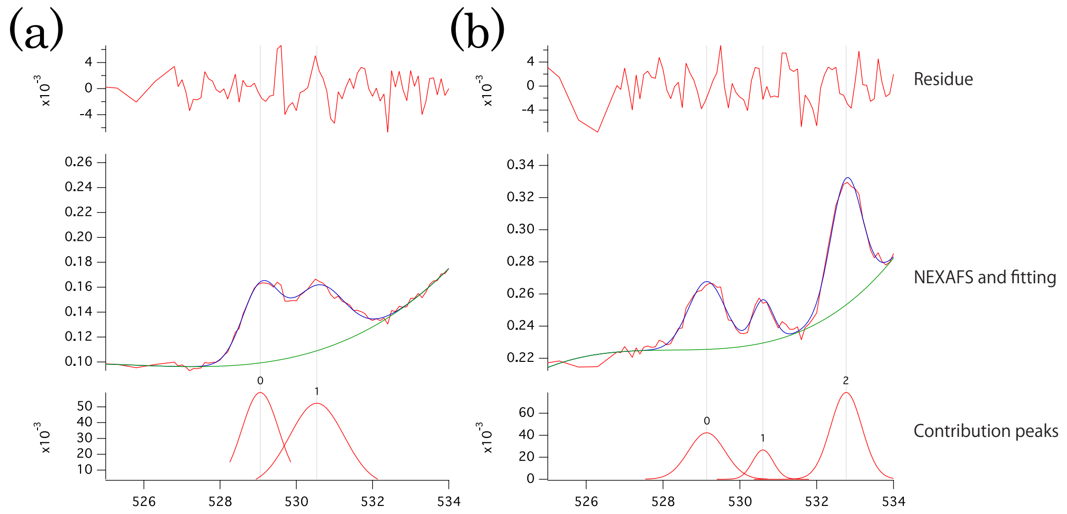

Figure S4. The red line of (a), and (b) are O-NEXAFS fitting results of cell, and sheath, respectively. Blue lines are fitting results. Green lines are backgrounds. Numbered peaks from 0 to 2 indicate contribution peaks in fitting process. Fitting parameter was concluded in Table S2.

Figure S5

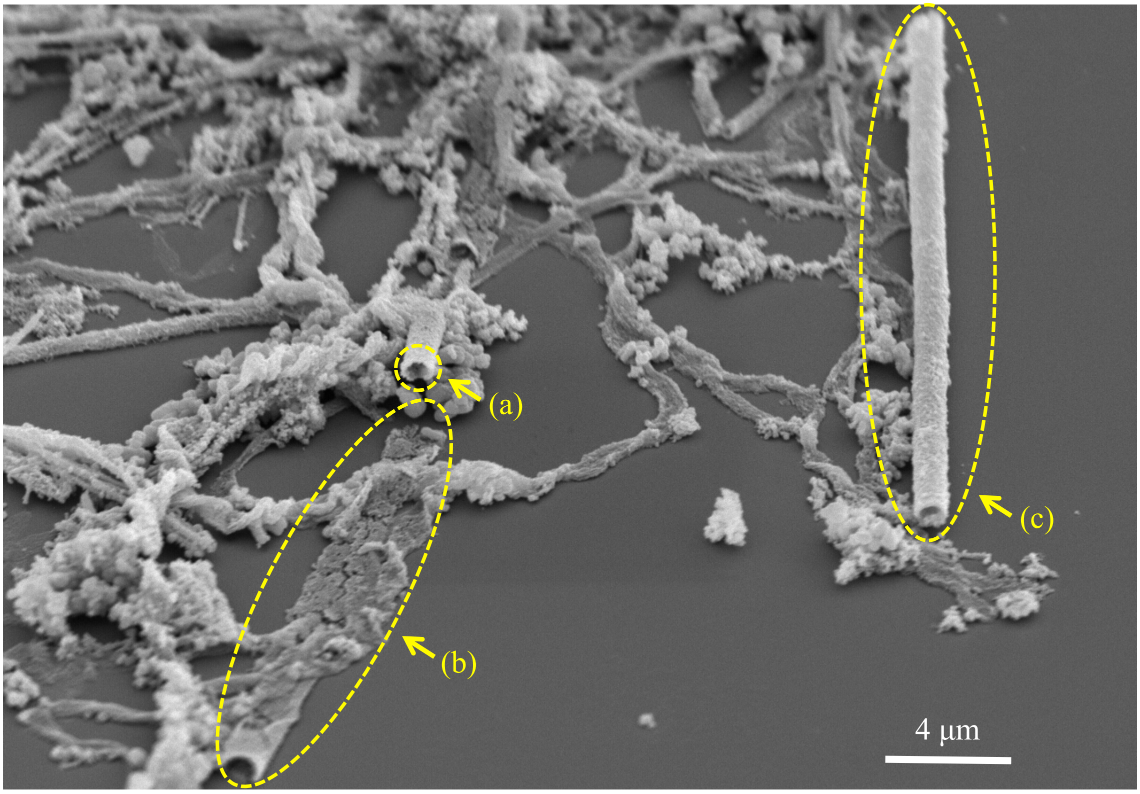

Figure S5. Secondly electron (SE) image of the Budo pond BIOS samples obtained from representative region. The sheath-like rod or long cylindrical shape stuffs and the stalk-like twisted fibers were identified from this image. Yellow circled region (a), (b), and (c) in the figure indicated that inner/outer globule-like structure (12, 30), inner globule-like structure on/in the burst sheath-like portion, and outer globule-like structure on the sheath-like portion, respectively.

Figure S6

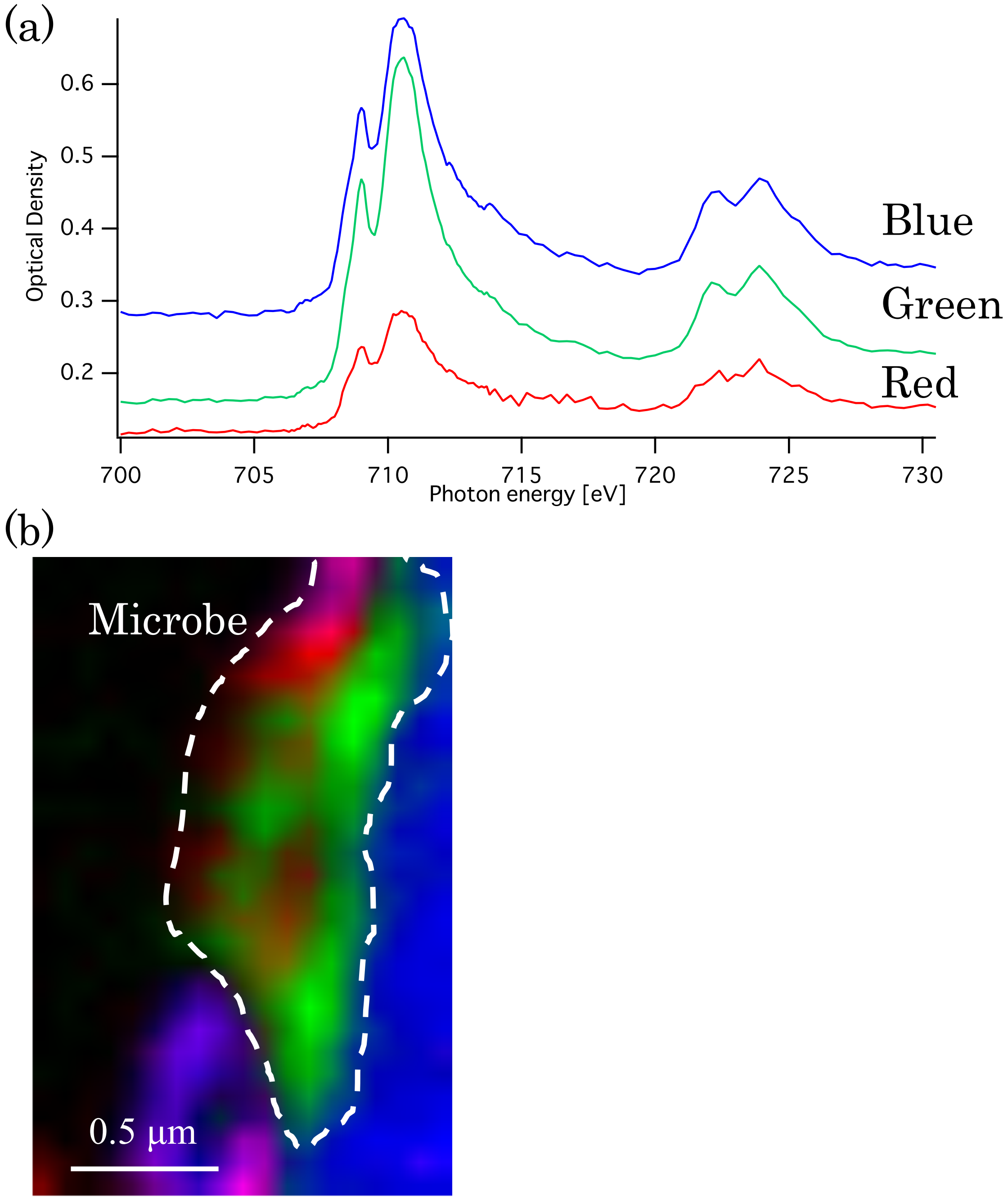

Figure S6. (a) Fe-NEXAFS extracted through PCA analysis using Mantis software. Three components (Red, Green and Blue) were obtained. (b) RGB map was composited using these three NEXAFS components through cluster analysis. Microbe is circled with a white dotted line. Red, green, and blue region correspond to cell, EPS, and sheath, respectively.

Figure S7

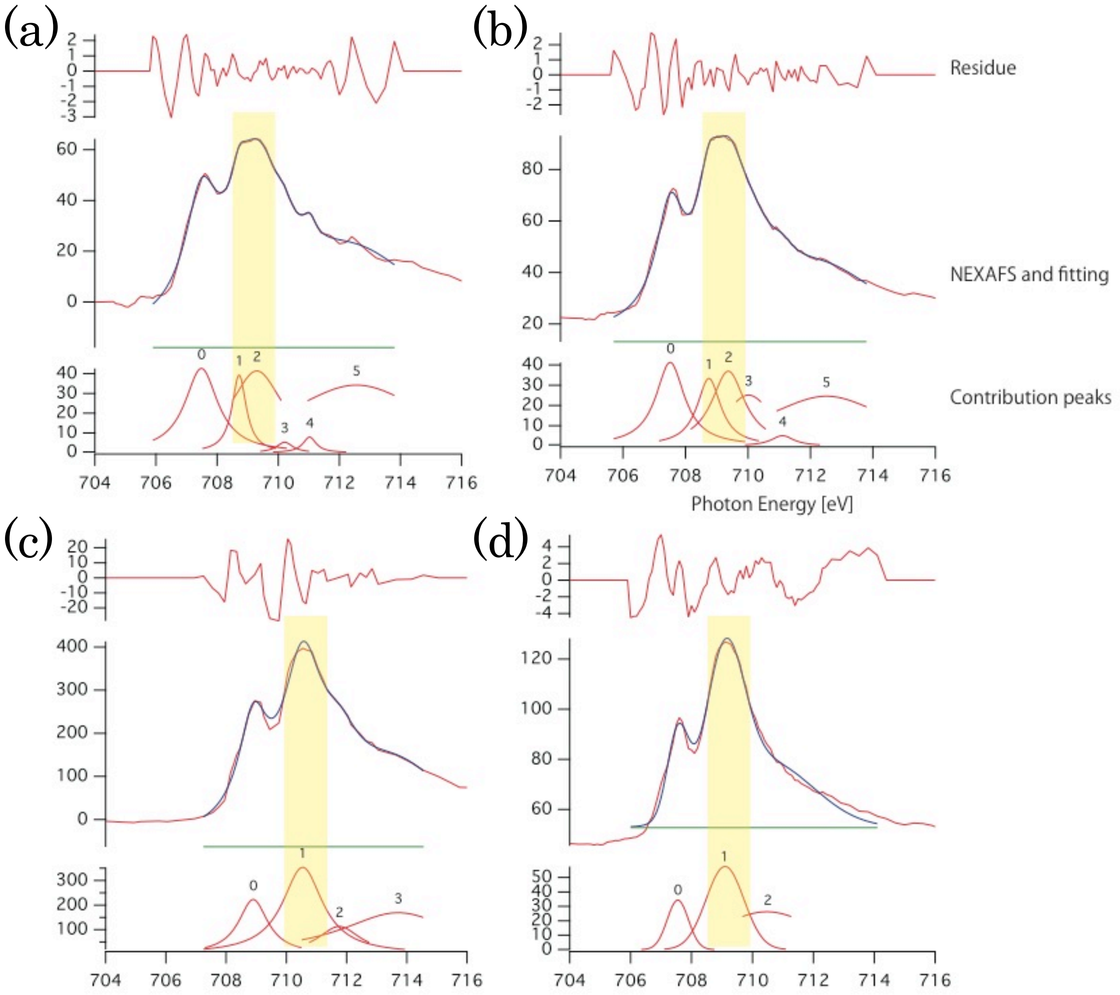

Figure S7. The red line of (a), (b), (c), and (d) are the Fe-NEXAFS fitting results of cell, EPS, sheath, and ferrihydrite, respectively. Blue lines are fitting results. Green lines are backgrounds. Numbered peaks from 0 to 5 indicate contribution peaks in fitting process. Yellow area is contributed to Fe(III) concentration.

Table S1. C-, N-, and O-NEXAFS transition energy table for major organic functional groups confirmed in standards and samples: table was summarized reports from reference of (2), (4-6), (18), (20-22), and (26-28).

**Carbon**	Peak Energy (eV)	Transition (bond)	Functional groups
	283.7-284.3	1s→π* (C=O)	Quinonic C=O
(I)	285.2	1s→π* (C=C)	Aromatic C (related to protein)
	286.5-287.2	1s→π* (C=O)	Ketonic C
(II)	287.3	1s→3p/σ* (C-OH)	Aliphatic C (dominant peak of lipid)
(III)	288.2	1s→π* (C=O)	Amide C (peptide bond of protein)
(IV)	288.6	1s→π* (C=O)	Carboxyl C (acidic polysaccharide carboxyl)
(V)	289.3	1s→3p/σ* (C-O)	O-alkyl C (dominant peak of DNA)
	290.2	1s→π* (C=O)	Carbonate

**Nitrogen**	Peak Energy (eV)	Transition (bond)	Functional groups
(I)	399.0	1s→π* (N=C)	Microbial nucleic materials (DNA)
(II)	399.9 (around 400.0)	1s→π* (N-C)	Microbial nucleic materials (DNA)
(III)	401.2-401.4	1s→π* (N-C=O)	Amide N (peptide bond of protein) with EPS and DNA
(IV)	402.2	1s→π* (N-C)	Amine/Amino N (C-N bond of protein) with EPS and DNA
	>405.0	1s→σ* (N-C)	†

**Oxygen**	Peak Energy (eV)	Transition (bond)	Functional groups
(I)	529.1-529.9	2p→3d (O-Fe)	O-Fe bond of iron oxides (related to Fe 3d e_g state)
(II)	530.5-531.3	2p→3d (O-Fe)	O-Fe bond of iron oxides (related to Fe 3d t_{2g} state)
(III)	532.1-532.7	1s→ π * (O=C)	Amide N (peptide bond of protein)
	>535.0	2p→4s, 4p/σ* (O-Fe/C)	O-Fe bond of iron oxides/mixing of O-C bonds of organics

† The broad feature observed at a higher energy (>405.0 eV) reflect to transitions of amino acid with mixing of aliphatic, alchol, carboxylic, and aromatic resonance (: protein with lipids, EPS, and DNA)

Table S2. The fitting parameters of Figure S4. (a) The t_{2g} peak height and peak area (in parentheses). (b) The e_g peak height and peak area (in parentheses). (c) Contribution peaks in the fitting energy region from 525.0 and 534.0 eV shown in Fig. S4. (d) e_g/t_{2g} ratio of height and area (in parentheses).

Sample	(a)	(b)	(c)			(d)
	t_{2g} height (area)	e_g height (area)	Contribution peaks from Fig. S4 (eV)			e_g/t_{2g} ratio height (area)
			529.1 (t_{2g})	530.5 (e_g)	532.7 (protein)	
Sheath	0.059 (0.072)	0.052 (0.093)	✓	✓		0.881 (1.576)
Cell	0.042 (0.050)	0.027 (0.018)	✓	✓	✓	0.628 (0.360)
